# DT-Web: a web-based application for drug-target interaction and drug combination prediction through domain-tuned network-based inference

**DOI:** 10.1186/1752-0509-9-S3-S4

**Published:** 2015-06-01

**Authors:** Salvatore Alaimo, Vincenzo Bonnici, Damiano Cancemi, Alfredo Ferro, Rosalba Giugno, Alfredo Pulvirenti

**Affiliations:** 1 Department of Mathematics and Computer Science, University of Catania, Viale A. Doria 6, Catania, Italy; 2 Department of Clinical and Experimental Medicine, University of Catania, Viale A. Doria 6, Catania, Italy; 3 Department of Computer Science, University of Verona, Strada le Grazie 15, Verona, Italy; 4 Correspondence can be also addressed to giugno@dmi.unict.it

**Keywords:** drug-target interaction, domain-tuned network-based inference, drug repositioning, drug combinations, drug substitutions, drug resistance, early stage analysis, online tool

## Abstract

**Background:**

The identification of drug-target interactions (DTI) is a costly and time-consuming step in drug discovery and design. Computational methods capable of predicting reliable DTI play an important role in the field. Algorithms may aim to design new therapies based on a single approved drug or a combination of them. Recently, recommendation methods relying on network-based inference in connection with knowledge coming from the specific domain have been proposed.

**Description:**

Here we propose a web-based interface to the *DT-Hybrid *algorithm, which applies a recommendation technique based on bipartite network projection implementing resources transfer within the network. This technique combined with domain-specific knowledge expressing drugs and targets similarity is used to compute recommendations for each drug. Our web interface allows the users: (i) to browse all the predictions inferred by the algorithm; (ii) to upload their custom data on which they wish to obtain a prediction through a *DT-Hybrid *based pipeline; (iii) to help in the early stages of drug combinations, repositioning, substitution, or resistance studies by finding drugs that can act simultaneously on multiple targets in a multi-pathway environment. Our system is periodically synchronized with *DrugBank *and updated accordingly. The website is free, open to all users, and available at http://alpha.dmi.unict.it/dtweb/.

**Conclusions:**

Our web interface allows users to search and visualize information on drugs and targets eventually providing their own data to compute a list of predictions. The user can visualize information about the characteristics of each drug, a list of predicted and validated targets, associated enzymes and transporters. A table containing key information and GO classification allows the users to perform their own analysis on our data. A special interface for data submission allows the execution of a pipeline, based on DT-Hybrid, predicting new targets with the corresponding p-values expressing the reliability of each group of predictions. Finally, It is also possible to specify a list of genes tracking down all the drugs that may have an indirect influence on them based on a multi-drug, multi-target, multi-pathway analysis, which aims to discover drugs for future follow-up studies.

## Background

In the last decades, pharmacology and therapeutic fields have encountered several development shortcomings due to prohibitive clinical costs related to novel drug discovery. The development of a new molecular entity is typically based on the process of discovering a new drug by modifying an existing one [1]. Recent trends in the pharmacogenomics area are going to exploit data mining and bioinformatics approaches, such as those based on drugs similarity, in connection with biological networks analysis. In Phatak et al. [2], a computational method for drug repositioning differs from previous similarity-based approaches since it combines chemical drug structures and drug target information computing similarity of drug target profiles via a bipartite-graph based approach. Bipartite graphs can also be used to provide a drug-target network for assessing the similarity between different disease inhibitors based on the connection to other compounds and targets. In this case, classical structure-based drug design and chemical-genomic similarity methods are combined with molecular graph theories. Another example of network based drug study is shown in Iorio et al. [3], where drug mode of action and drug repositioning are assessed using available gene expression profiles [4] to build a drug-drug network. The Connectivity Map databases [4] is a comprehensive reference catalog of genome-wide expression data from cultured human cells perturbed with many chemicals and genetic reagents, to connect human diseases with the genes that may cause them and drugs that can treat them. Literature-mining studies show that a large majority of new drugs bind to targets in some way related to a previously existing one [5-7]. A taxonomy and a comprehensive survey of new drugs discovery is contained in Csermely et al. [8].

An important role in the development of new drugs is given by the methods predicting drug-target interactions (DTI). Traditionally researchers have focused their attention on the development of drugs acting only on a specific protein family. On the other hand the more recent poly-pharmacology approach [9] combines actions of drugs and related multiple targets. The knowledge of these targets is fundamental to find out alternative applications (drug repositioning) as well as to identify side effects [10,11]. Despite all such efforts, today many interactions are still unknown and *in situ *experiments are too costly and time-consuming to be used as the sole strategy.

Various techniques to solve such a problem have been proposed [6,12-16]. In particular, the naive application [17] of the recommendation algorithm developed in Zhou et al. [18] has shown extremely promising results. In Alaimo et al. [19] an extension of the above method with the addition of domain-tuned knowledge led to the definition of the DT-Hybrid algorithm, combining bipartite networks projection and network resources transfer. The process is driven by 2D drug structural similarity, and target sequential similarity. The basic idea is that structurally similar drugs tend to have analogous behavior in similar proteins.

Another DTI prediction software is STITC*H 4.0 *[20-23]. It is a database of multi-species protein-chemical interactions, which, not only integrates many experimentally validated and manually curated data sources, but also provides a technique to compute predictions based on text-mining in research articles and analysis of chemical structures. The database combines this information to get an overall score that indicates the degree of confidence of the interaction. However, it can only be applied to the data in their internal database, and users can only search within such data or download the entire list of predictions, rather than providing their own compounds and/or proteins. Moreover, STITC*H 4.0 doe*s not compute drugs targeting combinations.

In addition, nowadays, research has been focused on new strategies exploiting metabolic and signaling networks since they show properties for the treatment of infection and cancer diseases. Graph searching algorithms help to exploit such data [24-29]. A metabolic pathway describes a series of chemical reactions occurring within a cell, in which enzymes catalyze the modification of an initial metabolite to form another product. A pathway may include biochemical reactions, complex assembly, transport events, catalysis events and physical interactions involving metabolites, namely proteins, DNA, RNA, small molecules and complexes. The set of metabolic pathways forms the metabolic network of a cell, which is part of its complex system, together with signaling pathways, representing the response to external signals, and other physical processes. Enzymes catalyzing a single reaction are usually essential [30], however related metabolites are frequently involved in several different pathways. Consequently, analyzing metabolic networks helps to identify interesting entities, such as: metabolites used as disease biomarkers [31,32]; or choke points (reactions that consume or produce a certain metabolite) [33,34], whose inhibition may cause lethal deficiencies or toxic accumulation of metabolites. Motivated by the success of manual pathway analysis, a pathway-based approach was proposed in Li et al. [35-37] where a causal drug-to-disease network is built by taking advantage of expert-curated biological knowledge including drug targets, pathways and disease downstream genes. Since, many human diseases cause metabolic deficiencies, the enzymes involved in such essential reactions can be considered promising drug targets [38-41]. Due to the complexity of diseases, the development of multi-target drugs [42,43] or drug combinations may be considered crucial. The choke point analysis, the comparison of metabolic networks of pathogenic and non-pathogenic strains and the load point analysis, may improve effective combinations prediction. These commonly consist in the identification of nodes having a high ratio of incident k-shortest paths [44,45]. On the other hand, it has been shown that co-targeting of crucial pathway points [46-49] is efficient against drug resistances both in anti-infective [50] and anti-cancer [51,52] strategies. Two relevant examples are RAS and Survivin associated diseases.

The RAS oncogenic mutations occur in human cancer quite frequently [53]. Therapeutic approaches based on the inhibition of RAS-mediated signalling have resulted ineffectively mainly due to the drug's toxicity. On the other hand, mutations of RAS, cause high levels of drug resistance. To overcome such shortcomings, Nussinov et al. [54] suggests an approach, inspired by Holzapfel et al. [55], based on drug combinations on parallel pathways.

The Survivin protein is linked to multiple pathways of cellular homeostasis [56]. These seem to confer to cancer cells a great plasticity, proliferation capability and resistance to death. Moreover, a large number of molecules, regulators, transcriptional networks and modifiers are directly or indirectly involved in Survivin related networks. Therefore, approaches considering Survivin in isolation and based on single-protein inhibitors [57] appear to be unsatisfactory. The development of drugs based on pathways inhibitors [58] exploiting the connectivity maps of Survivin to multiple signaling circuits [4] may result in a more effective approach.

An important application of biological network analysis concerns drug repositioning, a central task in new treatments discovery [13,14,59,60]. In Jahchan et al. [61] and Smith et al. [62], a pathway enrichment analysis in combination with gene expression profiles to explore new applications of existing drugs has been exploited. In Pan et al. [63], sixteen FDA-approved drugs were studied in order to understand their clinical functions through pathway analysis. Targets interacting or affected by the investigated drugs were extracted by mining public databases. Pathways having a co-occurrence of such targets were ranked through a p-value. Although directly and indirectly correlated drug targets were identified, important limitations arose due to relevant pathway retrieval and unknown targets finding.

In this paper, we propose DT-Web a software resource accessible via web at [64], using known sources, such as DrugBank [65-67] and PathwayCommons [68], in connection with DT-Hybrid [19] recommendation algorithm. It implements two analysis tools relying on networks: the first one uses a bipartite drug-target interaction network to predict novel high confidence DTIs, while the second one uses a multi-drug, multi-target, multi-pathway approach to guide the early stages of experimental analysis in drug combinations studies. In fact, even if researches can relay on public-domain databases of drug combination references, such as Liu et al. [69], there is still a lack of systematic computational approaches, and often several possible drug combinations are disclaimed by expert knowledge and verified via clinical trials. Motivated by the success of network-based approaches, our multi-purpose pathway analysis aims to provide a limited set of candidate drugs both for drug repositioning and combination, that can be directly evaluated by the experts or combined with other methods.

## Construction and content

The core of DT-Web has been developed in R and Java, while its front-end consists of a web interface developed in PHP with the backing of a MySQL database to store persistent information. We collected only drugs, targets and pathways for the *Homo Sapiens *species, and designed our methodology accordingly. DT-Web offers two major functionalities: (i) Drug-Target Interaction Prediction: the integration of *Drug-Bank *with *DT-Hybrid *to provide a comprehensive database of drugs, and their interactions with proteins (targets, enzymes, transporters, or carriers), either experimentally validated or predicted by DTI network inference; (ii) Multi-Purpose Pathway Analysis: the integration of *DrugBank, DT-Hybrid*, and *Pathway-Commons *to aid the experimental phase in drug combination studies by searching for drugs simultaneously acting on multiple targets.

### Drug-target interaction prediction

In order to extend our knowledge base, predictions are not limited to small molecules as in Alaimo et al. [19] rather extended to biotech drugs using an appropriate similarity measure based on their synthetic amino-acidic sequence, if available.

In order to search for alternative predictions, DT-Web allows uploading users own data. These may be processed by a *DT-Hybrid *based pipeline. Statistically reliable results will be returned. To guarantee data safety DT-Web assigns to each prediction a random identifier, which is required to retrieve the outcome. More precisely DT-Web actions are summarized below:

#### Drug-target interactions prediction pipeline

*Step 1*. Through an appropriate form, users can upload a DTI network equipped with a similarity matrix for each pair of drugs and targets. *DT-Hybrid *tuning parameters can be freely modified when this is requested.

*Step 2*. Users data are then checked to avoid incorrect format, and the *DT-Hybrid *algorithm is applied to obtain an initial list of predictions for each drug.

*Step 3*. Each target in the DTI network is mapped to an Entrez identifier in order to annotate each node with a set of GO terms. Next, for each pair of terms, we compute a similarity measure based on the node distance in the ontology DAG (Directed Acyclic Graph). Notice that, such a DAG has been previously modified to assign a value to each distance by connecting all the root nodes in the original DAG to a new single dummy root node.

*Step 4*. Finally, for each drug-target predicted pair, we calculate a correlation measure as the maximum similarity between ontological terms of validated targets and predicted ones. Then, for each drug, we select subsets of predicted targets with increasing minimum correlation, computing, each time, a p-value using a hyper-geometric distribution. Such subsets are filtered to return only those minimizing p-values in each drug. The p-values are computed as follows. Let *M *(*i, j*) be the *j*-th subset of predicted targets for drug *i, m *be the number of targets and *q *(*i, j*) be the number of targets having a correlation greater than the minimum computed in *M *(*i, j*). The p-value, p (*M *(*i, j*)), is the probability of drawing by chance |*M *(*i, j*)| = *k *(*i, j*) terms whose correlation is greater than the observed minima. An hyper-geometric distribution is used to compute such a value as:

$$p(M(i,j))=\frac{\left(\begin{array}{c} q(i,j)\\ k(i,j) \end{array}\right)\left(\begin{array}{c} m-q(i,j)\\ k(i,j)-k(i,j) \end{array}\right)}{\left(\begin{array}{c} m\\ k(i,j) \end{array}\right)}=\frac{\left(\begin{array}{c} q(i,j)\\ k(i,j) \end{array}\right)}{\left(\begin{array}{c} m\\ k(i,j) \end{array}\right)}$$

The p-value is used to provide a quality score to the association between predicted and validated targets of a single drug. No multiple test correction is applied, as each reported p-value is considered independent from the others.

### Multi-purpose pathway analysis

The aim is to discover the minimal set of drug targets that are able to affect a user-specified set of genes in a multi-pathway environment. The distances among such targets and user genes are limited to a given range in order to minimize drug side effects. The set of validated drug targets is extended with the *DT-Hybrid *predictions, along with their score to give a measure of confidence on each prediction.

The implemented pipeline has been divided into two main phases. The first phase is performed off-line and kept up-to-date whenever the DT-Web database is synchronized with the latest version of *DrugBank*. The second one is performed on-line and responses to the pasting of a list of genes through the submission form.

#### Off-line database building pipeline

The calculation of a multi-pathway environment requires huge computational resources and it is a time-consuming task. Because of this, the construction of such an environment, consisting of merging the Hom*o sapiens metaboli*c and signaling pathways contained in Reactome [70] and PID [71], is done off-line through a proprietary Java module, and stored in our database. The steps are the following.

*Step 1*. We retrieve all pathways by downloading *BioPax*[72] level 3 *XML *files from the *Pathway-Commons *web service, using PC2 [73] for the remote connection to the public database.

*Step 2*. For each pathway, we first normalize entity names if these exist (i.e. symbolic names for proteins, such as BRCA1), otherwise we consider the BioPax entity reference IDs.

*Step 3*. Subsequently, we collapse all nodes representing the same biological entity (protein sub-units or the same protein in different cell locations) in a single node, and we map them to the *DrugBank *database. Edge directions are kept as they are in the input network, except for those which connect a complex to a constituent protein that are made undirected.

*Step 4*. The entire set of retrieved pathways is merged into a single global network by mapping nodes and edges using their names and interaction types, respectively.

*Step 5*. Finally, to control the combinatorial explosion of such data, we store only directed shortest paths between proteins that lie at a distance of, at most, 9 edges. Moreover, since a path could contain edges belonging to different pathways, we decided to store for each edge the list of pathways where it appears. We, also, store the mapping to *DrugBank *database computed at step 3.

#### Real-time prediction pipeline

*Step 1*. The user provides a list of genes (names in HGNC format, or Uniprot Accession Number, or Entrez Gene Id, or HGNC Id, or Ensembl Gene Id) through a web interface. He can also set the ranges (min/max) for distances between drug targets and user-provided genes Direct-Indirect Range, or between each pair of drug targets Pair Range.

*Step 2*. Users' data are thus filtered to remove all proteins that are not present in our database. If the filtered list is not empty, a search is performed in our multi-pathway environment. The task selects all proteins which are at distance within the Direct-Indirect Range specified by the user.

*Step 3*. Each protein is then mapped in *DrugBank*, and those targeted by at least one drug are selected as a preliminary list of targets. Such a list is further filtered by removing all pairs of targets which are outside of the user-specified Pair Range.

*Step 4*. Next, by applying Chvatal et al. [74], we quickly compute an approximation of the minimum list of targets needed to reach all the user-specified genes.

*Step 5*. Finally, the list of all targets calculated in step 3, and each associated drug (experimentally validated or predicted), is returned to the user, along with the minimum set computed in step 4.

## Utility and discussion

DT-Web is an effective and user-friendly system that provides a web interface to visualize information on drugs and predicted targets, and to simplify the earlier experimental phases of drug combination studies by applying a multi-drug, multi-target, multi-pathway approach. All this is provided along with a constantly updated database containing the main and most reliable information from *DrugBank*. In addition, with the ability to upload their own data, our software does not limit the user to use only pre-computed information.

We, also, compared the prediction scores generated by *STITCH 4.0 *and DT-Web using a set of validated interactions taken from DrugBank. The analysis showed that the knowledge (i.e. experiments, structures, text-mining) given as input to *STITCH 4.0 *generate a distribution of scores which resulted bimodal and piled either on a low or a up range. On the other hand, DT-Web, which predictions scores are based on structure similarities and network inferences showed a Gaussian behavior. This difference is justifiable by both the kind of input knowledge and inference methods the two systems use.

**Figure 1:**
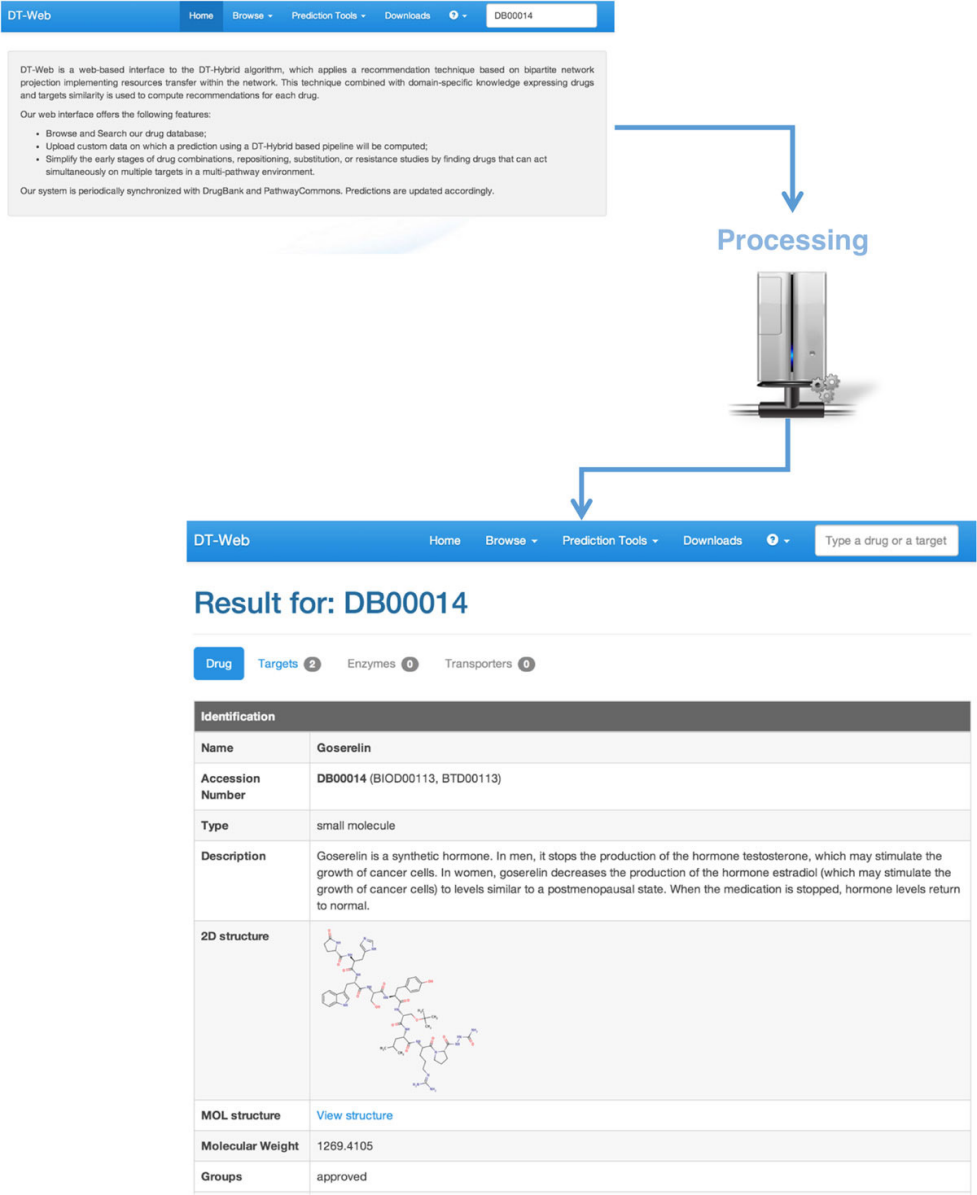
**DT-Web Search Example**. Once the user provides a query (either a part of the name or the accession number of a drug/target), DT-Web finds all the matching records in the internal database and returns them to the user in a page containing all the information requested. If all the records are drugs, the user will also see, if available, their two-dimensional structures, and a list of all targets, enzymes, transporters, carriers, and predicted targets, which have been computed through *DT-Hybrid*. Here user searched for *DB00014*

To use the integrated database, user provides one or more keywords (see our website [64] for a more detailed list of searchable information). The results will be returned on a page, which, as described in Figure 1, contains a list of the main information extracted from *DrugBank*, experimentally verified targets, enzymes, transporters, and carriers (along with papers providing such experimental evidence), and the predictions calculated by *DT-Hybrid*.

**Figure 2:**
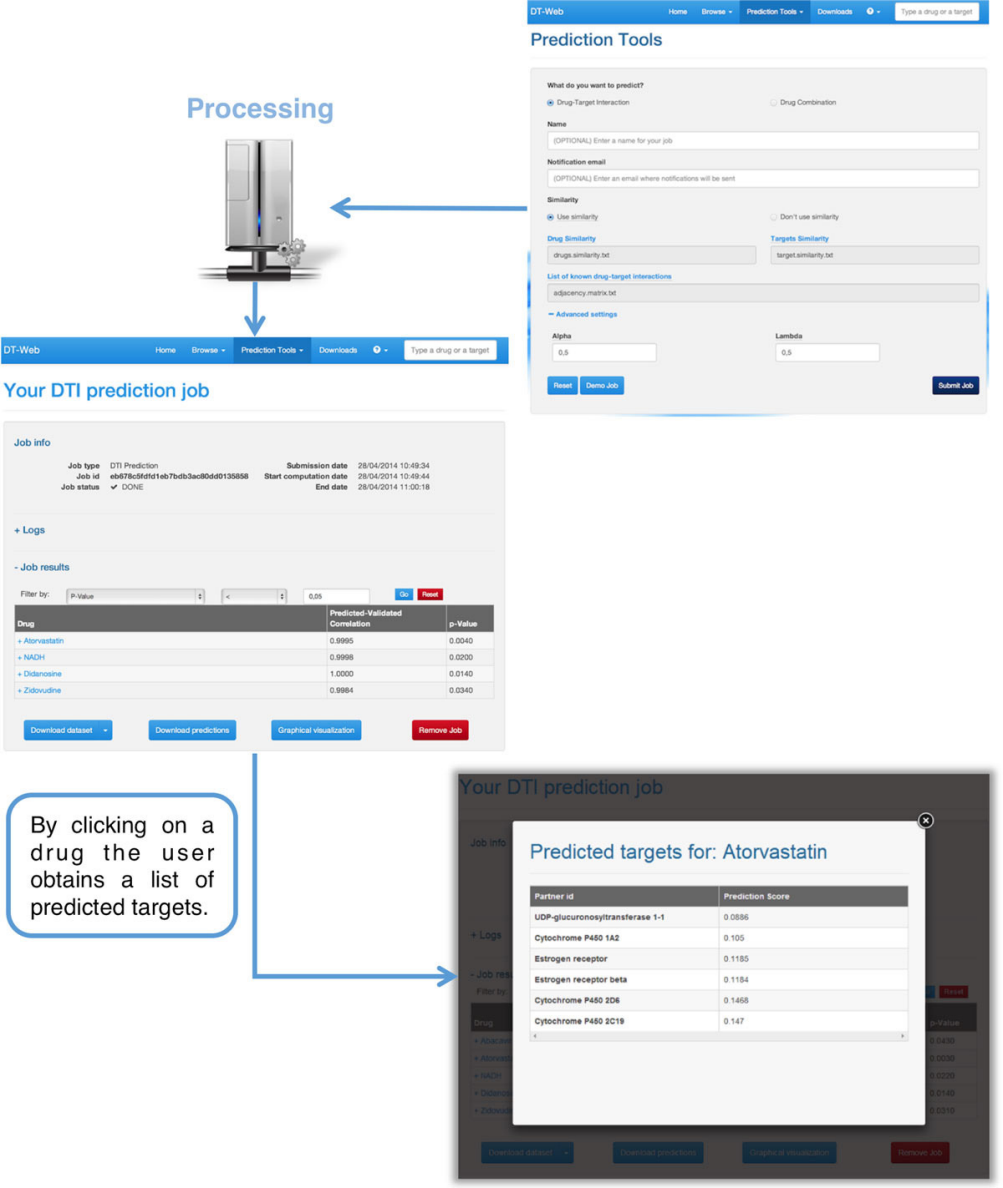
**DT-Web DTI Prediction Example**. Once the user provides its own DTI network and, if possible, two similarity matrices for each pair of drug and target, DT-Web applies the pipeline described above, after checking the validity of its input. At the end of such an operation, the user will see a page containing a list of all the drugs for which a result was available, along with the corresponding measures of correlation and p-value. By selecting one drug, the user will also see the list of all the predicted targets along with the scores assigned to each prediction by *DT-Hybrid*.

**Figure 3:**
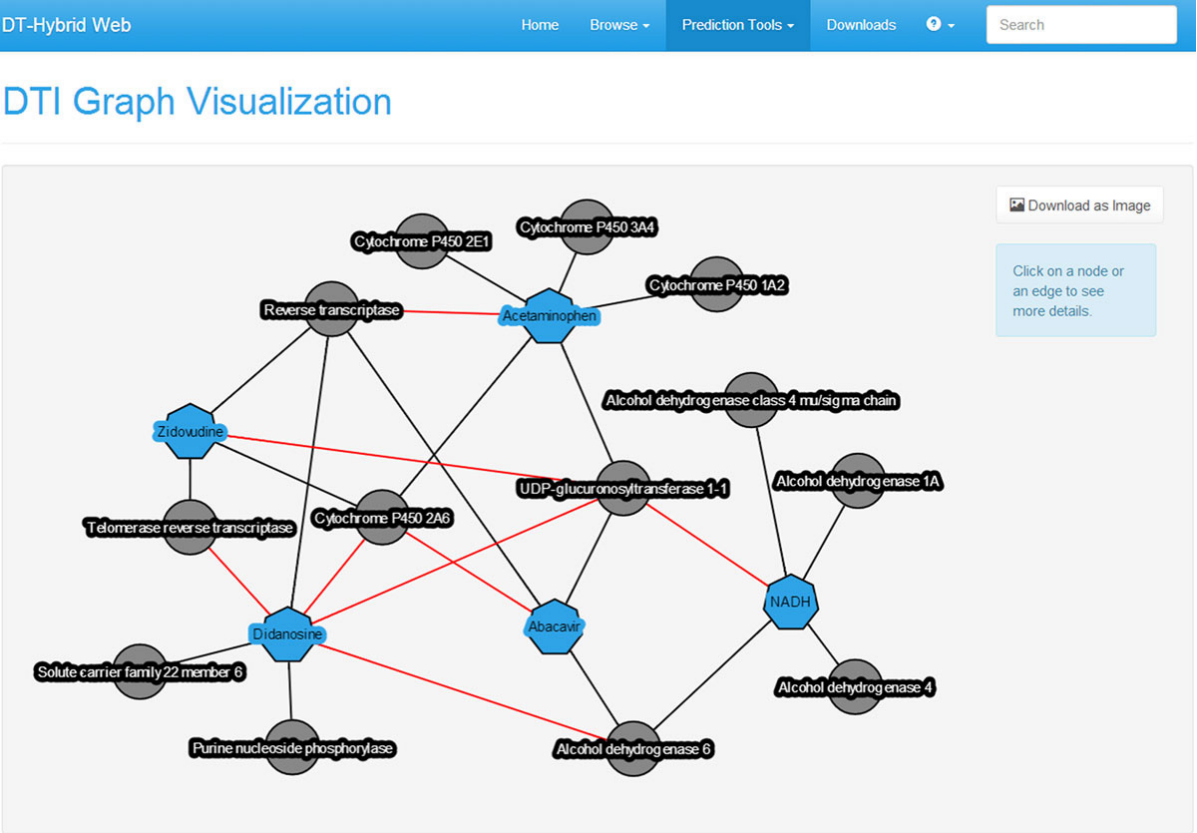
**DT-Web DTI network view**. A DTI network after applying our prediction pipeline. Each node represents a drug (blue heptagon) or a target (gray circle), while each edge represents a drug-target interaction (user-provided ones in black, predictions in red).

To take advantage of the DTI prediction pipeline on his own data, the user can choose the "Drug-Target Interaction" job in the "Prediction Tools" menu of our web interface. He will be able to upload his own data (format information and examples are available on our website [64]), which, after checking to ensure that the format is correct, will be analyzed with our pipeline. When data is submitted, DT-Web automatically generates a temporary page (see Figure 2) that allows to check the job status and, when ready, view results. To avoid long waits, an e-mail address, where a notification will be sent as soon as the results are available, can be provided. When viewing predictions, the user can apply filters based either on p-value or correlation by choosing an appropriate threshold. He will also be able to download a file containing all the results in text format, or visualize a graphical representation of the resulting DTI network (see Figure 3).

**Figure 4:**
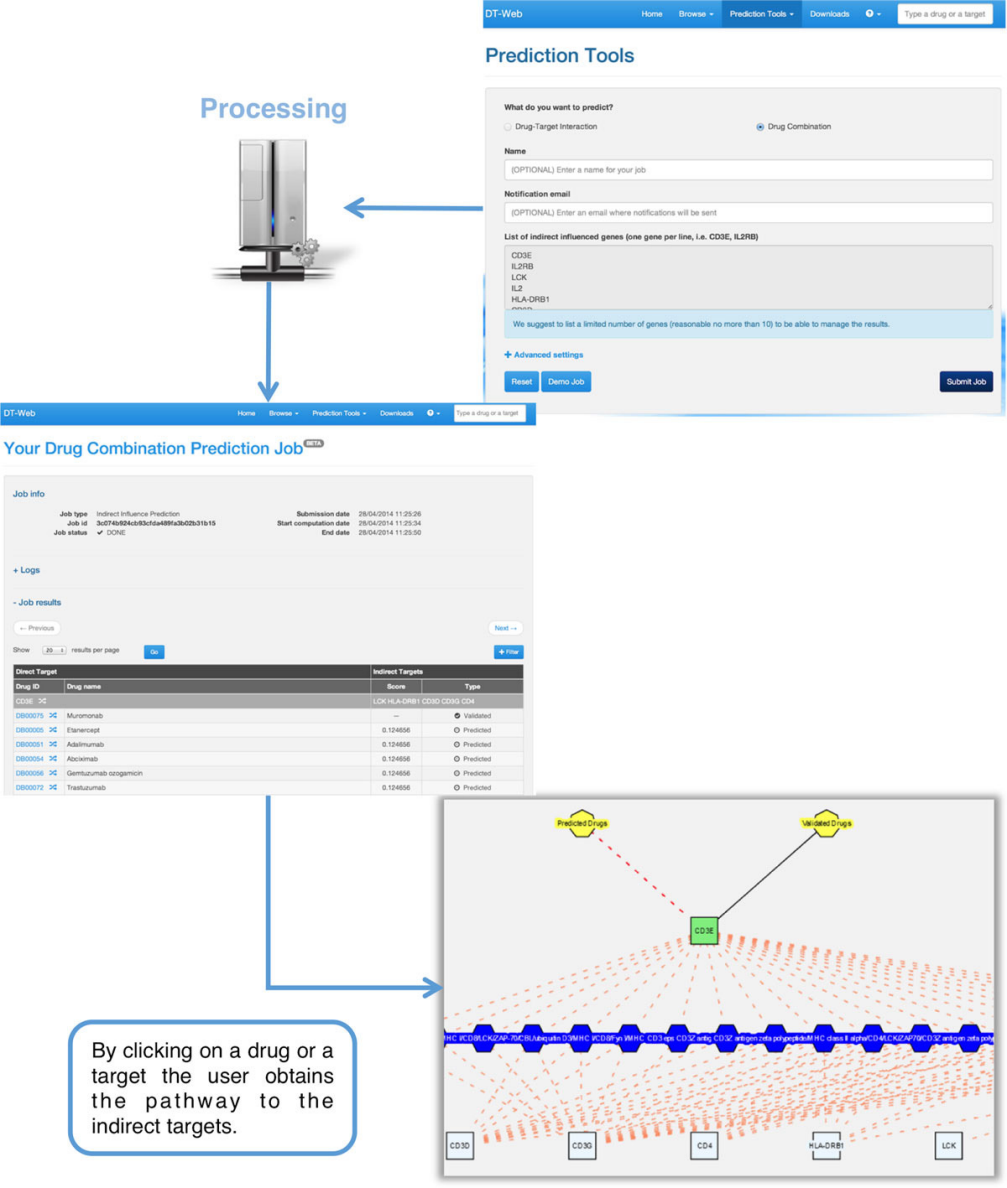
**DT-Web Drug Combination Prediction Example**. Once the user submits a list of genes, DT-Web calculates, using the pathways stored in the database, a list of possible drugs that indirectly target such genes, using the pipeline described above. When finished, the user will see a list of such drugs, ordered by direct and indirect targets, which can be filtered by selecting an appropriate combination of parameters (either one or more drugs/targets). By selecting a drug or a target, the user will also view an excerpt of the pathway used by the algorithm to compute the prediction. By clicking on a drug the user obtains a list of predicted targets.

To take advantage of our multi-purpose pipeline, the user can choose the "Drug Combination" job in the "Prediction Tools" menu. There, he can run the enriched analysis by indicating a set of indirectly influenced genes. Once the analysis starts, DT-Web automatically generates a temporary page (see Figure 4) that allows to check the job status and, if ready, view results. Once the analysis is performed, the results page will show the list of reachable targets and their interacting drugs. For each drug-target pair the *DT-Hybrid *prediction score is shown if the interaction does not come from a validated source. A list of filters can be applied to the results. The list of found drugs can be filtered by selecting only some of them as well as the drug-target interaction type, validated or predicted. Moreover, it is also possible to apply a filter on the set of found targets. For each target and drug, the user can visualize an interactive sub-network centered on the given entity. Additional information is available by clicking on the visualized elements, for example, the list of residing pathways can be visualized by clicking on each edge.

### Use cases

Users access DT-Web via the web interface where drugs and targets can be retrieved using a simple search engine. Furthermore, users can provide their own data to compute DTI predictions or simplify the early development stages in drug combination or repositioning studies. The algorithm is also available for download under Creative Commons license. This allows users to use DT-Web for large-scale studies, not suitable for our web environment. Examples illustrating our DTI prediction and multi-purpose pipelines follow.

*DT-Hybrid *has been used, in connection with other algorithms, to associate Simvastatin and Ketoconazole drugs to breast cancer treatment [75]. Our multipurpose pipeline, being a new software, has not yet been used for the intended studies. That is why we used our methodology to predict the combination of Propofol and Sevoflurane whose additive action produces consciousness and movement to skin incision during general anesthesia [76]. Both drugs interact with the GABA_A_ receptor. Propofol is a potentiator of the *β*2 subunit (GABRB2) of *GABA_A_*, while Sevoflurane is an agonist of the α1 subunit (GABRA1) of GABA_A_ with its binding site between both subunits [77]. Probably is such a location which hinders agonist activity, thereby producing mutually substitutable actions [78].

## Conclusions

The prediction of novel drug-target interactions is a fundamental process in order to reduce the costly and time-consuming phases of drug discovery and design. As a matter of fact, knowing the possible unknown effects on the proteome of a drug can be extremely useful in understanding its true potential or predicting side effects.

Other studies, such as drug repositioning, drug combinations or substitutions, help to eliminate the need to develop new drugs. Drug repositioning studies exploit existing drug for new purposes that go beyond the original ones, while drug combination studies try to modify or intensify the overall effect of two or more drugs by administering them together. Similarly, drug substitutions studies try to replace drugs having significant side effects with others aiming to reduce them. For this purpose, a tool that guides the early stages of the experimental process can significantly reduce the time and costs associated therewith.

This is the context of DT-Web. Its main goal is to provide a simple system that allows a user to quickly browse predictions of probable novel DTI, to produce new ones from their own data, or to simplify the experimental studies described above. All this by using a database obtained combining a new valuable resource *DT-Hybrid *with data extracted from *Drug-Bank *and *PathwayCommons*. Finally, all the results can be downloaded in text format or viewed on-line through the help of interactive graphical interfaces that simplify their understanding.

## List of abbreviations used

**DTI**: Drug-Target Interactions

**PID**: The pathway interaction database

**DAG**: Directed acyclic graph

## Competing interests

The authors declare that they have no competing interests.

## Authors' contributions

RG and AP conceived, developed and coordinated the research. SA and VB designed and developed the system. DC contributed to implementation aspects. SA, VB, AF, RG and AP analyzed results and wrote the paper.

## Declarations

Publication of this article has been funded by PON 2007-2013 grant, SIGMA - PON01 00683 - CUP B61H11000380005.

This article has been published as part of *BMC Systems Biology *Volume 9 Supplement 3, 2015: Proceedings of the Italian Society of Bioinformatics (BITS): Annual Meeting 2014: Systems Biology. The full contents of the supplement are available online at http://www.biomedcentral.com/bmcsystbiol/supplements/9/S3.
